# Is Time to Reach EDSS 6.0 Faster in Patients with Late-Onset versus Young-Onset Multiple Sclerosis?

**DOI:** 10.1371/journal.pone.0165846

**Published:** 2016-11-01

**Authors:** Raed Alroughani, Saeed Akhtar, Samar Ahmed, Raed Behbehani, Jasem Al-Hashel

**Affiliations:** 1 Division of Neurology, Department of Medicine, Amiri Hospital, Sharq, Kuwait; 2 Neurology Clinic, Dasman Diabetes Institute, Dasman, Dasman, Kuwait; 3 Department of Community Medicine and Behavioural Sciences, Faculty of Medicine, University of Kuwait, Jabriya, Kuwait; 4 Department of Neurology, Ibn Sina Hospital, Kuwait City, Kuwait; 5 Department of Neurology and Psychiatry, Minia University, Minia, Egypt; 6 Department of Ophthalmology, Ibn Sina Hospital, Kuwait City, Kuwait; 7 Department of Medicine, Faculty of Medicine, Kuwait University, Jabriya, Kuwait; University of Oxford, UNITED KINGDOM

## Abstract

**Background & Objectives:**

Published natural history data on late-onset of multiple sclerosis are limited. We aimed to assess the risk of attaining EDSS 6.0 among patients with late-onset (> 40 years) MS (LOMS) and young-onset (18–40 years) MS (YOMS).

**Methods:**

This cross-sectional cohort study was conducted to identify LOMS and YOMS patients’ with relapsing remitting course at MS diagnosis. Time (years) to reach sustained EDSS 6.0 was compared between LOMS and AOMS patients. Cox proportional hazards model was used to evaluate the demographic and clinical predictors of time to EDSS 6.0 in these cohorts.

**Results:**

LOMS and YOMS cohorts comprised 99 (10.7%) and 804 (89.3%) patients respectively. Spinal cord presentation at MS onset was more common among LOMS patients (46.5% vs. 32.3%). The proportions of LOMS and YOMS patients reaching EDSS 6.0 during the follow-up period were 19.2% and 15.7% respectively. In multivariable Cox proportional hazards model, older age at MS onset (adjusted hazard ratio (aHR) = 3.96; 95% CI: 2.14–7.32; *p* < 0.001), male gender (aHR = 1.85; 95% CI: 1.22–2.81; *p* = 0.004) and spinal cord presentation at onset (aHR = 1.47; 95% CI: 0.98–2.21; *p* = 0.062) were significantly associated with shorter time to EDSS 6.0.

**Conclusions:**

LOMS patients attained EDSS 6.0 in a significantly shorter period that was influenced by male gender and spinal cord presentation at MS onset.

## Background

The onset of multiple sclerosis (MS) typically occurs between the ages of 20 and 40 years; however, a wide spectrum exists since patients outside the age range have been increasingly diagnosed. As the general population ages, the MS prevalence in older adults rises and approximately 20% of MS patients experienced their first symptoms after age 40.[1, 2] Since the diagnosis of MS in older patients presents unique challenges, few studies have studied the natural history of late onset MS (LOMS).[3, 4] The clinical presentation and course of LOMS seem to be different from those with young onset MS (YOMS), which may lead to possible misdiagnosis with other white matter diseases.[5] There were conflicting reports on the rate of disease progression in this particular group.[6, 7] It was suggested that LOMS was associated with a poor prognosis that might have implications for initiation of aggressive therapy.[3] Despite the increasing MS prevalence in the Middle East, published epidemiological data on LOMS patients are scarce.[8, 9] In this study, we aimed to assess the risk of reaching sustained EDSS 6.0 among LOMS and YOMS cohorts and to evaluate associated demographic and clinical predictors using techniques of time to event analysis.

## Patients’ Cohorts and Methods

This cross-sectional cohort study was conducted using data from Kuwait National MS Registry (KNMSR). Established in 2010, this registry accounted for nearly 98% of the MS patients diagnosed in Kuwait.[9] The registry included the neurology tertiary hospital and other peripheral hospitals that have neurology units and MS clinics. All patients had been assessed by neurologists experienced in MS diagnosis using the revised 2010 McDonald diagnostic criteria,[10] and classified either as relapsing remitting (RR), secondary progressive (SP), or primary progressive (PP) MS.[11] An initial and a follow-up assessment sheets were provided to all hospitals to be filled by the treating neurologists. Once entered in the registry, patients were followed prospectively on regular basis (at least one visit every 6 months as per the registry protocol irrespective of relapses) and their clinical data were updated in the registry. MS patients with a relapsing onset and who had at least two EDSS assessments (in order to assess for EDSS changes) were included in the study. Patients with pediatric-onset (< 18 years), primary progressive MS and clinically isolated syndrome (CIS) were excluded. Patients who had symptoms onset at age of > 40 years were classified as late-onset MS (LOMS) cohort while those who presented between 18 and 40 years of age were classified as young-onset MS (YOMS).

For both LOMS and YOMS cohorts, data on gender, presentation at onset, disease duration, number of relapses, expanded disability status scale (EDSS) scores [12], prior/ ongoing DMTs were extracted from the registry database on December 31, 2015. Symptoms at onset were classified as supratentorial, visual pathway, cerebellar/ brainstem and spinal in order to improve the understanding of the anatomical location and functional impact of the first symptomatic lesion. The main outcome was time to a sustained EDSS 6.0, defined as “intermittent or unilateral constant assistance required to walk about 100 meters with or without resting”. The EDSS score had to be confirmed by a subsequent visit (at least 6 months from the baseline entry) and sustained such that every subsequent disability score was EDSS 6 or higher.

### Data analysis

We assessed the risk of attaining EDSS 6.0 in LOMS and YOMS cohorts during follow-up. Baseline characteristics of both LOMS and YOMS cohorts were summarized as frequencies (%) or median with inter-quartile range (IQR) and compared using log-rank or Mann-Whitney tests as appropriate. Kaplan-Meier survival curves were used to compare the EDSS 6.0—free experience of LOMS and YOMS cohorts during the follow-up period. The variables significantly (*p* ≤ 0.15) related with time to EDSS 6.0 on univariable analysis were considered for inclusion in multivariable analysis. Multivariable Cox proportional hazards model was used to identify demographic and clinical factors significantly (*p* < 0.05) and independently associated with time to EDSS 6.0 in LOMS compared to YOMS cohort. The adjusted hazard ratios (HR) and their 95% confidence intervals (CI) were used to interpret the final model. The institutional research board (IRB) of Ministry Of Health approved data collection of the national MS registry. All patients gave their written informed consents for the use of their data for research purposes. The IRB approved the consent procedure.

## Results

MS Patients aged 18 years or older (n = 955) were identified from KNMSR. Patients with PPMS (n = 32) and with incomplete data (n = 20) were excluded. Of remaining 903 patients, LOMS and YOMS cohorts comprised 99 (10.7%) and 804 (89.3%) respectively. The median (IQR) age (years) at MS onset was 45.9 (42.1) among LOMS compared to 26.6 (22.7–30.8) among YOMS patients Table 1. Female gender was predominant in both the cohorts while family history of MS was more common in YOMS patients. Median (IQR) disease duration (years), and number of relapses were nearly similar in both LOMS and YOMS cohorts. Among LOMS patients, spinal cord (46.5%) and multifocal (12.1%) presentations at onset were more common than the corresponding figures of (32.3% and (6.7%) among YOMS patients.

**Table 1:** Demographic and clinical characteristics of multiple sclerosis (MS) patients with late-onset (LOMS) or young-onset (YOMS).

Variables		LOMS = 99	YOMS = 804	*p*-value
		Median (IQR), range n (%)	Median (IQR), range n (%)	
Gender ^a^				0.187
	Female	71 (71.7)	523 (65.0)	
	Male	28 (28.3)	281 (35.0)	
Age (years) at onset ^b^				**< 0.001**
	Median (IQR)	45.9 (42.1–47.7)	26.6 (22.7–30.8)	
	Range	40.1–59.7	18.1–40.0	
Family history (y/n) ^a^		10 (10.1)	124 (15.9)	0.160
Disease duration (years) ^b^				0.352
	Median (IQR)	6.8 (4.6–10.1)	6.8 (4.6–15.7)	
	Range	3.0–27.8	0.3–27.8	
Number of relapses ^a^				0.297
	Median (IQR)	2 (1–4)	2 (1–4)	
	Range	1–11	1–12	
Clinical presentation ^a^				
	Supratentorial	21 (21.2)	201 (25.0)	0.409
	Optic pathway	19 (19.2)	178 (22.1)	0.503
	Brainstem / Cerebellar	29 (29.3)	229 (28.5)	0.392
	Spinal cord	46 (46.5)	260 (32.3)	**0.005**
	Multifocal	12 (12.1)	54 (6.7)	0.051
Disease Modifying Therapies ^a^				0.377
	First line therapy*	41 (47.1)	317 (43.3)	
	Second line therapy**	39 (44.8)	318 (43.4)	
	Not on treatment	7 (8.0)	97 (13.3)	
EDSS Status ^a^				0.368
	< 6.0	80 (80.8)	678 (84.3)	
	≥ 6.0	19 (19.2)	126 (15.7)	
Time (years) to reach EDSS 6.0 ^b^				**0.001**
	Median (IQR)	6.5 (3.3–10.5)	12.8 (8.3–15.3)	
	Range	2.6–14.0	3.0–27.5	

^a, b^ Computation of *p* value is based on either with Chi-square test for categorical variables ^a^ or with Mann-Whitney test for quantitative variables ^b^ IQR: Interquartile range; EDSS: Expanded Disability Status Scale

* First line therapy: Interferon Beta, Glatiramer Acetate, Teriflunomide, Dimethyl Fumarate

** Second line therapy: Fingolimod, Natalizumab, Mitoxantrone, Cyclophosphamide, Rituximab

**Fig 1:**
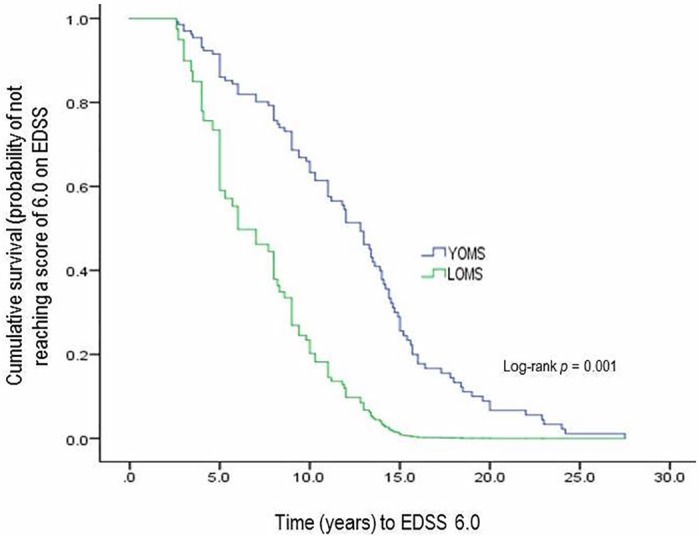
Kaplan-Meier survival curve i.e. probability estimates of not attaining a score of 6.0 on Expanded Disability Status Scale (EDSS) among cohorts with young-onset (YOMS) or late-onset (LOMS).

As expected, median age (years) at onset was significantly (*p* < 0.001) higher among LOMS (45.9; IRQ: 41.1–47.7) than YOMS (26.6; IRQ: 22.7.1–30.8) patients’ cohort. However median time (years) to EDSS 6.0 was significantly (*p* = 0.001) shorter in LOMS (6.5; IQR: 3.3–10.5) than YOMS (12.8; IQR: 8.3–15.3) patients (Fig 1). Non-parametric statistical comparison of median disease duration (years) and median number of relapses between LOMS and YOMS cohorts did not show statistically significant differences Table 1.

The proportions of LOMS and YOMS patients who reached EDSS 6.0 during follow-up were 19.2% and 15.7% respectively Table 2. When the disease course was analyzed, higher proportion of LOMS patients (26.3%; n = 26) converted to SPMS compared to YOMS patients (17.8%, n = 143). Furthermore, stratified on cohort type (LOMS/ YOMS), univariable analysis showed that male gender was significantly (*p* = 0.005) associated with time to reach EDSS 6.0. Although trending, no significant associations between time to attain EDSS 6.0 and brainstem/ cerebellar (*p* = 0.087) or spinal cord (*p* = 0.065) presentations at diagnosis were observed. However, family history of MS, optic pathway, spinal cord or multifocal presentations at onset, and use of disease modifying therapies had statistically non-significant associations with time to EDSS 6.0 on univariable analyses Table 2.

**Table 2:** Univariable associations of categorical demographic and clinical variables with time to reach EDSS 6.0 among multiple sclerosis (MS) patients stratified on late-onset (LOMS) or young-onset (YOMS) of disease.

Variables	Variables	LOMS cohort (N_1_ = 99)		YOMS cohort (N_2_ = 804)		Log-rank *p* -value
		Patients who reached EDSS 6.0, n_1_ = 19 (19.2%)	Category total	Patients who reached EDSS 6.0, n_2_ = 126 (15.7%)	Category total	
Gender						**0.005**
	Female	14 (73.7)	71	70 (44.4)	523	
	Male	5 (26.3)	28	56 (55.6)	281	
Family history		3 (16.7)	10	19 (15.1)	124	0.179
Clinical presentation						
	Supratentorial	2 (14.3)	21	28 (22.2)	201	0.800
	Optic	3 (15.6)	19	27 (21.4)	178	0.252
	Brainstem/Cerebellar	7 (36.6)	29	33 (26.2)	229	0.087
	Spinal cord	13 (68.4)	46	49 38.9)	260	0.065
	Multifocal	4 (21.1)	12	9 (7.1)	54	0.588
Disease Modifying Therapies						0.977
	Not on treatment	1 (5.6)	7	19 (17.0)	97	
	First line therapy*	11 (61.1)	41	36 (32.1)	317	
	Second line therapy**	6 (33.3)	39	57 (50.9)	318	

EDSS: Expanded Disability Status Scale

* First line therapy: Interferon Beta, Glatiramer Acetate, Teriflunomide, Dimethyl Fumarate

** Second line therapy: Fingolimod, Natalizumab, Mitoxantrone, Cyclophosphamide, Rituximab

Final multivariable Cox proportional hazards model showed the variables independently and significantly associated with shorter time to EDSS 6.0 were age (LOMS vs. YOMS) at MS onset (adjusted HR = 3.95; 95% CI: 2.14–7.32; *p* < 0.001), male gender (adjusted HR = 1.85; 95% CI: 1.22–2.81; *p* = 0.004) and spinal cord presentation at MS onset (adjusted HR = 1.47; 95% CI: 0.98–2.21; *p* = 0.062). Other demographic variables and/ or clinical symptoms were not significantly associated with time to EDSS 6.0 in multivariable analysis Table 3.

**Table 3:** Multivariable Cox proportional-hazards model of variables associated with time to reach EDSS 6.0 in cohorts of patients with late-onset (LOMS) or young-onset (YOMS) of disease.

Variable	Un-adjusted hazard ratio	95% Confidence interval	Adjusted hazard ratio	95% Confidence interval	*p*-value
Cohort type (LOMS/ YOMS)	3.49	1.91–6.37	3.96	2.14–7.32	**< 0.001**
Gender (male/female)	1.61	1.07–2.43	1.85	1.22–2.81	**0.004**
Spinal cord presentation (y/n)	1.51	1.01–2.26	1.47	0.98–2.21	0.062

## Discussion

MS symptoms on initial presentation at age 40 years or more may be considered as red flags, since MS commonly presents between ages 20–40 years. Nevertheless, with better diagnostics in terms of MRI detection of typical demyelinating lesions in the brain and spine, patients with late onset of typical demyelinating symptoms and signs have been increasingly diagnosed with MS. In our cohort, 11% of the analyzed patients had their disease onset after age 40. The age cutoff was arbitrary in most of the previous reports.[7, 13–17] Some authors used the cutoff of 50 or 60 years of age. We believed that MS onset beyond the typical age (20–40) is considered a late presentation, and since natural history data showed that the onset of PPMS and SPMS often clustered around age 40,[18, 19] we have chosen the this specific cutoff. A recent study showed that despite the difference in the relapse rate prior to progressive disease, most relapsing patients converted to SPMS at similar age (mean 39.7 years).[20] Based on the available literature, the proportion of LOMS patients was commonly subtracted from the adult MS population. The reported LOMS rates ranged from 3.4% to 12.7% depending upon the cutoff points for age at the onset in most of the hospital or clinic based studies.[3, 4, 7, 14, 17] This wide variations in the proportions of LOMS patients in most studies are often attributed to use of different study designs, cohort sizes investigated, case ascertainment procedures, and referral bias. Other factors such as life expectancy in the studied population, and genetic susceptibility may play important roles in studies assessing older patients. The MS diagnosis in older patients is challenging especially in patients with known vascular risk factors such as hypertension and diabetes wherein white matter lesions may be attributed to ischemia rather demyelination. A longitudinal follow-up of such patients may help in establishing the diagnosis especially in those with progressive course.[6] It is also probable that the proportion of LOMS patients is underestimated in various studies given the precautionary measures in diagnosing older patients with demyelinating disorders.

Although the female to male ratio in LOMS patients has been generally reported to be similar to younger MS patients,[7, 15, 21] we found a higher F: M ratio (2.5:1) than those reported by other studies ranging from 1.4:1 to 1.7:1.[3, 7, 15] The exclusion of PPMS cohort with its male predominance from our cohort may partially explain this difference. Furthermore, the increasing MS incidence among females during the last decade in this and other settings might have partially contributed to this female preponderance among LOMS patients.

In our study, LOMS had significantly shorter time to reach EDSS 6 compared to younger patients with MS. LOMS patients had 3.6 times the risk of attaining EDSS 6.0 compared to YOMS during the follow-up period. Similar results were reported by a registry-based study conducted at the University of British Columbia, Canada that showed a tendency for faster rate of disease progression in older patients reaching EDSS 6 compared to YOMS (16.7 versus 27.7 years, *p* < 0.0005).[4] In a study analyzing several outcomes of disease progression including EDSS 6.0 step in 1609 relapsing /445 progressive onset MS patients, several age cut-offs (including 40–49 year and ≥ 50 years) were correlated with EDSS milestones. It was shown that the median times from clinical onset of MS to EDSS 6 decreased with increasing age at onset (median from 29.0 to 9.0 years, *p* < 0.0001).[22]

There is no universally accepted definition for secondary progressive MS (SPMS) to date; SPMS is mostly diagnosed in retrospect, based on a history of gradual worsening after an initial relapsing disease course.[23] In an attempt to define SPMS with high accuracy and feasibility, a prospective observational cohort study tested several candidate definitions. Among the patients with ≥ 5 years follow-up after the SPMS diagnosis, 78% showed a positive disability trajectory and 70% reached an EDSS of ≥ 6 at censoring.[24] Others tried to measure the disease progression index using the EDSS score as a numerator and disease duration as a denominator.[7, 25] Kis and colleagues pair-matched 52 LOMS with 52 YOMS patients on gender and disease duration and reported a higher disease progression index in LOMS cohort (0.60 vs. 0.35).[7] Another study reported slightly higher disease progression index in LOMS patients, when compared with non-LOMS patients (0.88 versus 0.37; *p* < 0.0001).[25] Thus, EDSS 6.0 has been shown to be indirectly associated with disability progression. In a study included 1023 patients, MS onset at age 40 doubled the risks of developing secondary progression compared to age 20.[26] This risk was independent of disease duration and early relapse frequency. On the other hand, prospective data suggested that older age is the only linear predictor with a strong relationship to greater disability.[27] With advancing age, repair, remyelination, and other physiological functions become less robust.[28] This could be explained by intrinsic characteristics that pre-determine maturation and aging of oligodendrocyte precursor cell irrespective of extrinsic factors, and epigenetic changes known to occur during aging.[29]

In our cohorts, spinal cord presentation at onset was significantly more prevalent among LOMS (46.5%), which was consistent with other studies.[7, 30] Kis et al found higher spinal presentation at onset in LOMS patients (81% vs. 48%; *p* = 0.024).[7] Indirectly, Cossburn et al. described the association of lower limb motor symptoms, sphincteric and sexual dysfunction with older patients.[30]

In a multivariable analysis, spinal cord presentation at onset was associated with an increased the risk of attaining EDSS 6.0. Studies on the long-term consequences of spinal cord presentations in relapsing LOMS are limited. One retrospective series that evaluated spinal MS concluded that the progression seemed to be mainly due to age at onset rather than the site of lesion.[31] Spinal cord lesions were associated with higher risk of conversion to clinically definite MS and the presence of two or more focal spinal lesions were independently associated with a higher risk of conversion to MS independent of brain lesions in two prospective CIS cohorts.[32, 33]

Occasionally, a clinico-radiological paradox may be evident when there is a lack of association between spinal symptoms/ signs and a detectable spinal lesion on MRI. Improvement in MRI techniques has allowed a better assessment of correlation between the clinical and radiological parameters. Oh et al. found that cord atrophy had a good correlation with spinal lesion count and disease progression in 124 patients stratified according to EDSS score. MRI measures were more abnormal in the high- vs. low-disability subgroup of patients with high lesion counts (*p* < 0.05).[34] Similarly, Bernitsas et al. studied 150 patients (RRMS = 93; SPMS = 57) and found a significant correlation (r = 0.75, *p* < 0.0001) between cervical cord atrophy and EDSS progression.[35] These findings support the concept that quantitative MRI measures have the ability to provide clinically relevant information beyond that which may be gleaned from measures of MRI lesion load alone.[34]

Additionally, we found that male gender was associated with higher risk of attaining EDSS 6.0. In a large international register studying 14453 relapse-onset patients, it was shown that males progressed significantly faster in their EDSS than females (0.133 versus 0.112 per year; *p* < 0.001,).[36] Similarly, Leray et al, observed that the time to reach DSS 6 from clinical onset of MS was shorter in males than in females (median16.0 vs. 20.0 years, *p* <0.0001), despite nearly similar mean age at MS clinical onset among both males and females patients.[22] A more rapid attainment of EDSS scores of 4, 6 and 7 from MS onset were shown to be higher in male patients in other studies.[37, 38]

Several limitations of this study need to be taken into account while interpreting the results of this study. First, most (67.7%) of the clinical data of patients who had disease onset or being diagnosed before the establishment of the registry in 2010 was retrospectively collected which might have resulted in recall bias in some of the collected data such as relapses. Second, the lack of MRI data on our patients for analysis are usual deficiencies in most observational registries. Third, higher proportion of LOMS patients was on first line therapy compared to YOMS, which precludes definitive interpretation of the effect of disease modifying therapies on disease progression. If substantiated with data from clinical trials or large prospective longitudinal studies, our findings may have reassuring clinical implications for treatment selection and disease monitoring in patients with late-onset MS. Fourth, although the designated cutoff age of LOMS were carefully chosen based on a clear rationale, they may still be regarded as arbitrary given the lack of prior studies determining the most appropriate cutoff ages for patients presenting beyond the typical age of onset. Additionally, the ability to distinguish between individuals with high/low disability in our study is limited by the constraints of the EDSS, and is thus heavily weighted toward ambulatory disability. Nevertheless, this was a population-based rather than a hospital/clinic based study and has a relatively adequate proportion of LOMS patients that allowed precise statistical comparison with patients of younger onset. Additionally, it is the first study in the Middle East, which assessed the LOMS pointing some key indicators of disease progression in the region.

In summary, patients with late onset MS tended to rapidly reach EDSS 6.0. Male gender and spinal symptoms at onset were associated with increased risk of attaining this disability measure. Since the prevalence of LOMS will continue to increase, there is a need to better understand the natural history of these patients and their response to earlier institution of treatment.
